# A toolkit for plant lipid engineering: Surveying the efficacies of lipogenic factors for accumulating specialty lipids

**DOI:** 10.3389/fpls.2022.1064176

**Published:** 2022-12-14

**Authors:** Yingqi Cai, Xiao-Hong Yu, John Shanklin

**Affiliations:** ^1^ Biology Department, Brookhaven National Laboratory, Upton, NY, United States; ^2^ Department of Biochemistry and Cell Biology, Stony Brook University, Stony Brook, NY, United States

**Keywords:** lipid engineering, fatty acid, triacylglycerol, lipid droplet, specialty fatty acid, specialty lipid, lipogenic factor

## Abstract

Plants produce energy-dense lipids from carbohydrates using energy acquired *via* photosynthesis, making plant oils an economically and sustainably attractive feedstock for conversion to biofuels and value-added bioproducts. A growing number of strategies have been developed and optimized in model plants, oilseed crops and high-biomass crops to enhance the accumulation of storage lipids (mostly triacylglycerols, TAGs) for bioenergy applications and to produce specialty lipids with increased uses and value for chemical feedstock and nutritional applications. Most successful metabolic engineering strategies involve heterologous expression of lipogenic factors that outperform those from other sources or exhibit specialized functionality. In this review, we summarize recent progress in engineering the accumulation of triacylglycerols containing - specialized fatty acids in various plant species and tissues. We also provide an inventory of specific lipogenic factors (including accession numbers) derived from a wide variety of organisms, along with their reported efficacy in supporting the accumulation of desired lipids. A review of previously obtained results serves as a foundation to guide future efforts to optimize combinations of factors to achieve further enhancements to the production and accumulation of desired lipids in a variety of plant tissues and species.

## Introduction

All organisms can convert carbohydrates into fatty acids (FAs), the building blocks of both phospholipids for membrane synthesis and triacylglycerols (TAGs) for carbon and energy storage. Some organisms including plants have evolved specialized lipogenic factors to accumulate large quantities of TAGs or produce specialty FAs. Bio-based TAGs, also known as storage lipids, contain more than twice the energy of carbohydrates, making them a sustainable energy-dense source of biofuels (Ohlrogge and Chapman, 2011; Singh et al., 2021). Specialty lipids containing high levels of specialty FAs can serve as feedstocks for jet fuel, nutraceuticals, and industrial products because of their distinct physical and functional properties (Dyer et al., 2008; Park et al., 2021). However, natural sources of these lipids are limited and therefore are not sufficient to meet growing demand. Plants use carbon and energy acquired from photosynthesis to synthesize FAs and accumulate TAGs and thus represent a renewable and economically viable platform for lipid production. General conservation of lipid synthesis across kingdoms makes it possible to engineer agronomic plants for the production and accumulation of desired lipids by inter-species heterologous expression of many lipogenic factors.

**Figure 1:**
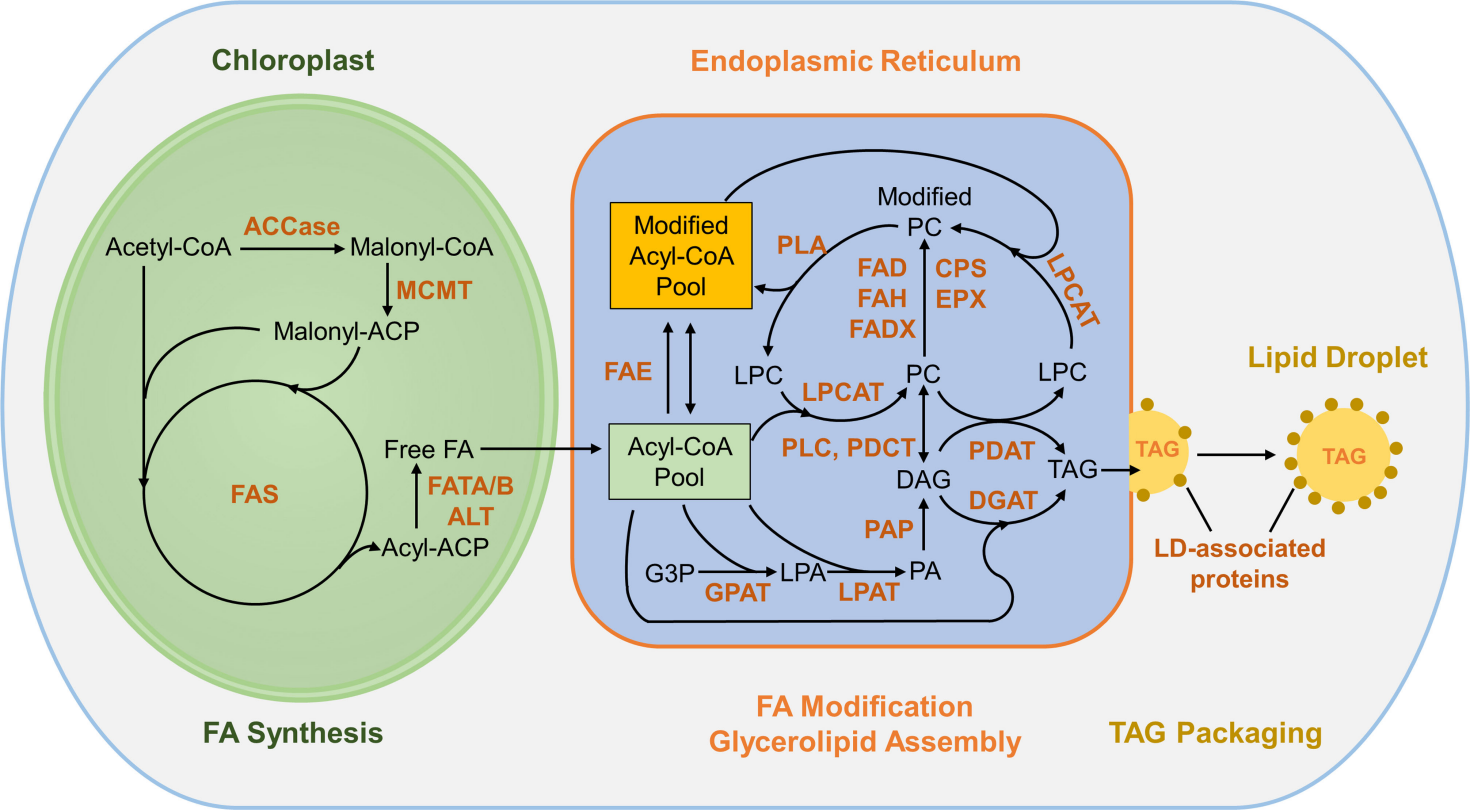
Overview of FA synthesis and TAG assembly in plants. The conversion of acetyl-CoA to malonyl-CoA by ACCase is the first committed step in fatty acid synthesis in plastids. With acetyl-CoA serving as the starting unit, the acyl chain is extended by sequential condensation of two-carbon units from malonyl-ACP by FAS complex. FAs exported from plastids enter the acyl-CoA pool in the ER and can be incorporated into PC, where acyl groups are modified and re-enter the acyl-CoA pool thorough acyl editing. The Kennedy pathway incorporates acyl-CoA into glycerolipids *via* sequential acylation of G3P by GPAT, LPAT and DGAT. TAG can be synthesized through acyl-CoA-dependent (DGAT converting DAG and acyl-CoA to TAG) and acyl-CoA-independent (PDAT synthesizing TAG from DAG and PC) pathways. TAGs are packaged into LDs and protected by LD-associated proteins. ACCase, acetyl-CoA carboxylase; ACP, acyl carrier protein; CoA, coenzyme A; MCMT, malonyl-CoA: ACP malonyltransferase; FAS, fatty acid synthase; FATA/B, fatty acyl thioesterase A/B; ALT, acyl-lipid thioesterase; FAE, fatty acid elongase; GPAT, glycerol-3-phosphate acyltransferase; LPAT, lysophosphatidyl acyltransferase; PAP, phosphatidic acid phosphatase; DGAT, diacylglycerol:acyl-CoA acyltransferase; PDAT: phospholipid:diacylglycerol acyltransferase; PLC, phospholipase C; PDCT, phosphatidylcholine:diacylglycerol cholinephosphotransferase; LPCAT, lysophosphatidylcholine acyltransferase; PLA, phospholipase A; CPS, cyclopropane synthase; EPX, epoxygenase; FAD, fatty acid desaturase; FAH; fatty acyl hydroxylase; FADX, fatty acid conjugase; G3P, glycerol-3-phosphate; LPA, lysophosphatidic acid; PA, phosphatidic acid; DAG, diacylglycerol; PC, phosphatidylcholine; LPC, lysophosphatidylcholine; TAG, triacylglycerol.

In plant cells, FAs are synthesized from acetyl-coenzyme A (CoA) in plastids (**Figure 1**; Ohlrogge and Browse, 1995; Li-Beisson et al., 2013). The heteromeric acetyl-CoA carboxylase (ACCase) catalyzes the conversion of acetyl-CoA to malonyl-CoA, the first committed step in FA synthesis. With acetyl-CoA serving as the starting unit, the acyl chain is extended by the FA synthase complex (FAS) through sequential condensation of two-carbon units from malonyl-acyl carrier protein (ACP). FAs reaching a certain chain length (typically C16 or C18) are released from ACP by fatty acyl thioesterases (FAT)A/B and exported from plastids. These FAs then enter the acyl-CoA pool in the endoplasmic reticulum (ER), where they are further modified and incorporated into glycerolipids (**Figure 1**). ER-localized FA elongase (FAE) can add additional two-carbon units to acyl-CoA to further elongate FAs. The acyl chains esterified to phosphatidylcholine (PC) undergo modifications to introduce double bond(s) and functional groups to FAs and the modified FAs re-enter the acyl-CoA pool through acyl-editing, a dynamic acyl exchange between PC and the acyl-CoA pool. TAGs can be assembled *via* sequential acylation of glycerol-3-phosphate (G3P) with acyl-CoA as the acyl donor catalyzed by glycerol-3-phosphate acyltransferase (GPAT), lysophosphatidyl acyltransferase (LPAT), phosphatidic acid phosphatase (PAP), and diacylglycerol:acyl-CoA acyltransferase (DGAT) (reviewed in Li-Beisson et al., 2013; Xu and Shanklin, 2016). Alternatively, PC can serve as the acyl donor for acylation of diacylglycerol (DAG) to form TAG by phospholipid:diacylglycerol acyltransferase (PDAT) (Dahlqvist et al., 2000). In addition, FAs esterified to PC may enter the TAG pool through the conversion of PC to DAG and subsequently to TAG by phosphatidylcholine:diacylglycerol cholinephosphotransferase (PDCT) or phospholipase C (PLC) (Wang, 2001; Lu et al., 2009). TAGs synthesized within the bilayer of the ER membrane are packaged into cytosolic lipid droplets (LDs) by coordination of LD-related proteins including SEIPIN, LDAP, LDIP, Oleosin, Caleosin, and Steroleosin (reviewed by Chapman et al., 2012 and Pyc et al., 2017).

In plants, most TAGs are synthesized and stored in seeds, serving as a major reserve of carbon and energy for seed germination and seedling establishment. In contrast, plant vegetative tissues usually contain trace amounts of TAGs despite their high capacity for FA synthesis. Studies of lipid engineering in plants increasingly focus on 1) producing value-added specialty lipids in seeds of oilseed crops (e.g., *Camelina sativa* and *Brassica napus*) by introducing specialized lipogenic factors to increase the value of seed oil (Xu et al., 2020; Yuan and Li, 2020) and 2) enhancing the accumulation of TAGs in vegetative tissues of high-biomass crops (e.g., *Nicotiana tabacum*, *Sorghum bicolor*, and *Saccharum* spp. *Hybrids*) by overexpressing TAG-enhancing lipogenic factors to increase the overall lipid yield on a per plant and unit land area basis (Vanhercke et al., 2019b; Park et al., 2021). Almost all these lipid engineering approaches require heterologous expression of lipogenic factors from other organisms. In this review, we present a survey of work evaluating the efficacy of lipogenic factors from various organisms in engineering lipids in selected target plant species. Specifically, we provide a list of lipogenic factors that exhibit specialized functional features in FA synthesis and modification (Section I and **Table 1**), glycerolipid assembly (Section II and **Table 2**) and LD biogenesis (Section III and **Table 3**) and highlight recent progress in optimizing combinations of lipogenic factors for enhanced production of desired lipids, and discuss challenges and future opportunities for lipid engineering in plants. It can be difficult to correlate the effects of expressed genes on lipid metabolism, especially those discussed in review articles, with their precise coding sequences. In this work we have included accession numbers wherever possible to address and remedy this issue.

**Table 1:** List of specialized lipogenic factors involved in FA synthesis and modification used for lipid engineering in plants.

Lipogenic Factor	Origin Species	Accession No.	Target Species	Effects of Heterologous Expression on Lipid Metabolism	Reference
FATB1	*U. californica*	M94159	*B. napus*	Produced seed oil containing up to 50% MCFA (lauric acid 12:0).	Eccleston et al., 1996; Voelker et al., 1996
			*A. thaliana*	Produced MCFA (lauric acid 12:0) accounted for up to 37% and 43% of seed oil in wild-type and *aae15/16* mutant backgrounds, respectively.	Tjellström et al., 2013
	*U. californica*	M94159 Q41635	*C. sativa*	Produced MCFA (lauric acid, 12:0 and myristic acid, 14:0) accounted for 21% of seed oil.	Kim et al., 2015b
	*C. viscosissima*	AEM72522	*C. sativa*	Produced MCFA (C8-C14) accounted for 15% of seed oil.	Kim et al., 2015b
	*C. pulcherrima*	AGG79283	*C. sativa*	Produced MCFA (myristic acid, 14:0) accounted for 1.6% of seed oil.	Kim et al., 2015b
FATB2	*C. palustris*	AAC49180	*A. thaliana*	Produced MCFA (myristic acid, 14:0) accounted for up to 39% and 42% of seed oil in wild-type and *aae15/16* mutant backgrounds, respectively.	Tjellström et al., 2013
			*C. sativa*	Produced MCFA (myristic acid, 14:0) accounted for 24% of seed oil.	Kim et al., 2015b
	*C. hookeriana*	AAC49269	*B. napus*	Produced MCFA (caprylic acid, 8:0; capric acid, 10:0; lauric acid, 12:0) accounted for up to 40% of seed oil.	Dehesh et al., 1996
			*A. thaliana*	Produced MCFA (caprylic acid, 8:0; capric acid, 10:0) accounted for up to 22% and 25% of seed oil in wild-type and *aae15/16* mutant backgrounds, respectively.	Tjellström et al., 2013
	*C. hookeriana*	AAC49269	*C. sativa*	Produced MCFA (C8-C14) accounted for 12.6% of seed oil.	Kim et al., 2015b
FATB3	*C. pulcherrima*	KC675178	*A. thaliana*	Produced MCFA (caprylic acid, 8:0; capric acid, 10:0) accounted for up to 6% and 12% of seed oil in wild-type and *aae15/16* mutant backgrounds, respectively.	Tjellström et al., 2013
			*C. sativa*	Produced MCFA (C8-C14) accounted for 2.9% of seed oil.	Kim et al., 2015b
			*C. sativa*	Produced MCFA (myristic acid, 14:0) accounted for 7.5% of seed oil.	Kim et al., 2015b
ALT1	*A. thaliana*	NM_103226 At1g35290	*C. sativa*	Produced MCFA (lauric acid, 12:0 and myristic acid, 14:0) accounted for up to 3.5% of seed oil.	Kalinger et al., 2021
	*A. thaliana*	NM_103226 At1g35290	*N. benthamiana*	Produced approximately 50 nmol MCFA (lauric acid, 12:0 and myristic acid, 14:0) per gram leaf fresh weight.	Kalinger et al., 2021
ALT4	*A. thaliana*	NM_001334359 At1g68280	*C. sativa*	Produced approximately 1% MCFA (caproic acid, 6:0; caprylic acid, 8:0; capric acid, 10:0; and myristic acid, 14:0) in seed oil.	Kalinger et al., 2021
			*N. benthamiana*	Produced approximately 53 nmol MCFA (caproic acid, 6:0) per gram leaf fresh weight.	Kalinger et al., 2021
FATB2 KAS4	*C. hookeriana*	AAC49269 AF060519	*B. napus*	Increased MCFA by 30-40% in seed oil as compared to that of plants expressing *ChFATB2* alone.	Dehesh et al., 1998
FATB1 KAS4	*C. palustris* *C. hookeriana*	U38188 AF060519	*B. napus*	Increased MCFA by 40% in seed oil as compared to that of plants expressing *ChFATB2* alone.	Dehesh et al., 1998
FAH	*C. purpurea*	EU661785	*A. thaliana*	Produced hydroxy FA (ricinoleic and densipolic) up to 25% of seed oil in the Arabidopsis fad2/fae1 mutant.	Meesapyodsuk and Qiu, 2008
FAH12	*R. communis*	U22378	*A. thaliana*	Produced hydroxy FA (ricinoleic, densipolic, lesquerolic, and auricolic acids) accounted for up to 19% of seed oil in wild-type, *fad2/fae1*, *fad3*, and *fad3/fae1* plants.	Broun and Somerville, 1997; Smith et al., 2003
			*C. sativa*	Produced hydroxy FA to approximately 15% of seed oil in the wild-type background.	Aryal and Lu, 2018
FAH12-1	*H. benghalensis*	KC533767	*A. thaliana*	Produced up to 21% hydroxy FA in seed oil of the *fad2/fae1* mutant.	Zhou et al., 2013
FAH12-2	*H. benghalensis*	KC533768	*A. thaliana*	Produced up to 18% hydroxy FA in seed oil of the *fad2/fae1* mutant.	Zhou et al., 2013
EPX	*C. palaestina*	Y16283	*A. thaliana*	Produced epoxy FA accounted for up to 6.2% of seed oil, which was further increased to 21% when co-expressed with *CpFAD2* in the *fad3/fae1* mutant.	Singh et al., 2001; Zhou et al., 2006
			*G. hirsutum*	Produced epoxy FA accounted for 17% of seed oil when co-expressed with *CpFAD2*.	Zhou et al., 2006
	*E. lagascae*	AF406732	*N. tabacum*	Produced epoxy FA accounted for 15% of total FA in calli.	Cahoon et al., 2002
			*G. max*	Produced epoxy FA accounted for 8% of total FA in somatic embryos.	Cahoon et al., 2002
	*S. laevis*	AY462108	*A. thaliana*	Produced 2.4% epoxy FA (vernolic acid) in seed oil.	Hatanaka et al., 2004
			*P. hybrida*	Produced epoxy FA (vernolic acid) accounted for 0.5% of total lipids when transiently expressed in leaves.	Li et al., 2010
			*G. max*	Produced 8% epoxy FA (vernolic acid) in seed oil.	Li et al., 2010
	*V. galamensis*	N/A	*N. benthamiana*	Produced epoxy FA accounted for 8.7% of total leaf lipids, which was further increased to 13.1% when co-expressed with *VgFAD2*.	Sun et al., 2022
FADX	*M. charantia*	AF18252	*G. max*	Produced conjugated FA (eleostearic and parinaric acids) accounted for up to 18% of total FA in somatic embryos.	Cahoon et al., 1999
			*A. thaliana*	Produced eleostearic acid accounted for approximately 13% of total seed FA in the *fad3/fae1* mutant.	Cahoon et al., 2006
	*I. balsamina*	AF182520	*G. max*	Produced conjugated FA (eleostearic and parinaric acids) accounted for up to 5% of total FA in somatic embryos.	Cahoon et al., 1999
	*C. officinalis*	AF310156	*G. max*	Produced calendic acid accounted for approximately 22% of total FA in somatic embryos.	Cahoon et al., 2006
			*A. thaliana*	Produced calendic acid accounted for approximately 15% of total seed FA in the *fad3/fae1* mutant.	Cahoon et al., 2006
	*V. fordii*	AF525535	*A. thaliana*	Produced eleostearic acid accounted for approximately 6% of total seed FA in the *fad3/fae1* mutant.	Cahoon et al., 2006
			*A. thaliana*	Produced approximately 2% eleostearic acid in leaf neutral lipids.	Yurchenko et al., 2017
Δ^9^-AAD	*A. syriaca*	U60277	*A. thaliana*	Failed to produce detectable ω-7 FA in seed oil.	Bondaruk et al., 2007
Δ^9^-AAD	*D. unguis-cati*	AF051134	*A. thaliana*	Produced approximately 28% and 9% ω-7 FAs in Arabidopsis and Brassica seed oil, respectively.	Bondaruk et al., 2007
Com25 (mutated Δ^9^-AAD)	*R. communis*	N/A	*A. thaliana*	Resulted in accumulation of ω-7 FAs to 14% and 56% of seed oil when expressed in wild-type and fab1/fae1 backgrounds, respectively.	Nguyen et al., 2010
			*C. sativa*	Increased the content of ω-7 FAs to approximately 17% of seed oil.	Nguyen et al., 2015
SnD9D AnD9D	*S. nodorum* *A. nidulans*		*A. thaliana*	Produced ω-7 FAs accounted for approximately 24% of seed oil and further increased the level of ω-7 FAs to up to 71% of seed oil when co-expressed with Com 25 in fab1/fae1 seeds.	Nguyen et al., 2010
Com25 FAT5	*R. communis* *C. elegans*		*C. sativa*	Produced ω-7 FAs accounted for approximately 23% and 65% of seed oil in wild-type and fab1/fae1/fatb backgrounds, respectively.	Nguyen et al., 2015
CPS	*S. foetida*	AF470622	*A. thaliana*	Produced a trace amount of CPA (~0.05% of total FA) in seeds of the *fad2/fae1* mutant.	Yu et al., 2011
	*E. coli*	M98330	*N. benthamiana*	Produced up to 3.7% CPA in total FA in leaves, which was increased to 11.8% when *NbFAD2* was silenced, and a novel C18:2CPA.	Okada et al., 2020
	*E. coli*	944811	*A. thaliana*	Produced substantial amounts of CPA (up to 9.1% of total FA) in seeds of the *fad2/fae1* mutant.	Yu et al., 2014
			*C. sativa*	Produced up to approximately 10% CPA in total seed FA.	Yu et al., 2018
CPS1	*G. hirsutum*	AY574036	*A. thaliana*	Produced detectable amounts of CPA (up to 1% of total FA) in seeds of the *fad2/fae1* mutant.	Yu et al., 2011
			*N. benthamiana*	Produced up to 1% CPA in total FA in leaves, which was increased to 4.8% when *NbFAD2* was silenced.	Okada et al., 2020
Δ^12^-DES Δ^15^/ω^3^-DES Δ^6^-DES Δ^6^-ELO Δ^5^-DES Δ^5^-ELO Δ^4^-DES	*P. sojae* *P. infestans* *O. tauri* *P. patens* *T.* sp. *O. tauri* *O.* RCC809	EGZ11023 XP_002902599 XP_003082578 AAL84174 AAM09687 CAI58913 JGI: 40461	*C. sativa*	Represent an optimal combination of genes for EPA and DPA biosynthesis in oilseeds. Routinely produced EPA and DHA in excess of 20% total seed oil.	Han et al., 2020; Han et al., 2022

N/A, not available.

**Table 2:** List of specialized lipogenic factors involved in glycerolipid assembly used for lipid engineering in plants.

Lipogenic Factor	Origin Species	Accession No.	Target Species	Effects of Heterologous Expression on Lipid Metabolism	Reference
LPAT	*C. nucifera*	U29657	*B. napus*	Enabled efficient MCFA (lauric acid, 12:0) deposition at the *sn*-2 position of TAG, resulting in accumulation of lauric acid to over 50% of seed oil when co-expressed with *UcFATB1*.	Knutzon et al., 1999
	*C. nucifera*	Q42670 U29657	*C. sativa*	Increased lauric acid (12:0) and myristic acid (14:0) in seed oil and at the *sn*-2 position of TAG when co-expressed with *UcFATB1* and *CpFATB2*, respectively.	Kim et al., 2015b
	*V. galamensis*	N/A	*N. benthamiana*	Increased the level of epoxy FA from 8.7% to 16.7% when co-expressed with *VgEPX*.	Sun et al., 2022
	*S. foetida*	KC894726	*A. thaliana*	Enriched CPA in glycerolipids and increased CPA accumulation up to 35% of total seed FA when co-expressed with *EcCPS* in the *fad2/fae1* mutant.	Yu et al., 2014
			*C. sativa*	Enriched CPA in glycerolipids and increased CPA levels up to 18% of total seed FA when co-expressed with *EcCPS* in the *fad2/fae1* mutant.	Yu et al., 2018
LPAT2	*R. communis*	EU591533	*A. thaliana*	Slightly increased the level of hydroxy FA in seed oil in the *fae1* mutant expressing *RcFAH12*.	Shockey et al., 2019
	*V. fordii*	MH823254	*A. thaliana*	Significantly increased eleostearic acid content in seed oil in the *fad3/fae1* mutant expressing *VfFADX*.	Shockey et al., 2019
	*C. viscosissima*	ALM22867	*C. sativa*	Enabled deposition of capric acid (10:0) at the *sn*-2 position of TAG.	Kim et al., 2015a
LPAT2a	*C. pulcherrima*	ALM22869	*C. sativa*	Enabled deposition of capric acid (10:0) at the *sn*-2 position of TAG.	Kim et al., 2015a
LPATB	*C. pulcherrima*	ALM22873	*C. sativa*	Enabled deposition of myristic acid (14:0) but not capric acid (10:0) at the *sn*-2 position of TAG.	Kim et al., 2015a
LPCAT	*V. galamensis*	N/A	*N. benthamiana*	Increased the level of epoxy FA from 8.7% to 19.4% when co-expressed with *VgEPX*.	Sun et al., 2022
PDCT	*R. communis*	EQ973818	*A. thaliana*	Enriched hydroxy FA in DAG and TAG, increased hydroxy FA levels to nearly 20% of seed oil when co-expressed with *RcFAH12* in the wild-type background, and partially restored the decreased seed oil content caused by *RcFAH12* expression.	Hu et al., 2012
	*L. chinensis*	KU926346	*C. sativa*	Enhanced the transfer of CPA from PC to DAG and led to a 57% increase in CPA accumulation in TAG when co-expressed with *EcCPS* relative to expressing *EcCPS* alone in the *fad2/fae1* mutant.	Yu et al., 2019
PLCL1	*R. communis*	XM_002523576	*C. sativa*	Enriched hydroxy FA in TAG, increased hydroxy FA levels to 22% of seed oil when co-expressed with *RcFAH12*.	Aryal and Lu, 2018
PDAT1A (PDAT1-2)	*R. communis*	NM_001323733	*A. thaliana*	Channeled hydroxy FA into TAG and increased hydroxy FA to 27% of seed oil when co-expressed with *RcFAH12* in the *fae1* mutant.	van Erp et al., 2011
DGAT1	*C. pulcherrima*	KU055625	*C. sativa*	Enriched MCFA (capric acid, 10:0) in TAG and increased capric acid content to 14.5% of seed oil when co-expressed with *CvFATB1*.	Iskandarov et al., 2017
	*V. galamensis*	EF653277	*P. hybrida*	Resulted in a 2-fold increase in epoxy FA in leaves co-expressing *VgDGAT1* and *SlEPX* relative to expressing *SlEPX* alone.	Li et al., 2010
			*G. max*	Increased the accumulation of epoxy FA to 15% of seed oil when co-expressed with *SlEPX*.	Li et al., 2010
	*C. ellipsoidea*	KT779429	*A. thaliana*	Increased seed oil content by 8–37%.	Guo et al., 2017
			*B. napus*	Increased seed oil content by 12–18%.	Guo et al., 2017
DGAT2	*R. communis*	EU391592	*A. thaliana*	Enhanced the incorporation of hydroxy FA into TAG and increased the level of hydroxy FA to approximately 30% of seed oil when co-expressed with *RcFAH12* in the *fae1* mutant.	Burgal et al., 2008; Shockey et al., 2019
	*V. galamensis*	FJ652577	*P. hybrida*	Resulted in a 6-fold increase in epoxy FA in leaves co-expressing *VgDGAT2* and *SlEPX* relative to expressing *SlEPX* alone.	Li et al., 2010
			*G. max*	Increased the accumulation of epoxy FA to 26% of seed oil when co-expressed with *SlEPX*.	Li et al., 2010
	*V. fordii*	DQ356682	*A. thaliana*	Redirected eleostearic acids from phospholipids to TAGs, increased eleostearic acid to approximately 12% of neutral lipids in leaves and mitigated the negative growth effects caused by *FADX* expression. No significant increase in eleostearic acid was observed in seeds.	Yurchenko et al., 2017; Shockey et al., 2019
	*M. musculus*	BC043447	*N. benthamiana*	Increased TAG contents by 20-fold when transiently expressed in leaves.	Cai et al., 2019
DGAT2-2	*C. esculentus*	N/A	*N. tabacum*	Increased TAG contents in leaves to 5.5% DW, which is 7.2-fold and 1.7-fold higher than that in wild-type leaves and leaves expressing *AtDGAT1*, respectively. Increased the proportion of oleic acid in leaf lipids.	Gao et al., 2021
DGAT5 (DGTT5)	*N. oceanica*	KY273672	*N. benthamiana*	Increased TAG contents by 2-fold when transiently expressed in leaves.	Zienkiewicz et al., 2017
			*A. thaliana*	Increased TAG contents by 6-fold in leaves and increased seed oil content by 50%.	Zienkiewicz et al., 2017
DGAT (DAcT)	*E. alatus*	GU594061	*A. thaliana*	Resulted in accumulation of acTAG up to 40% of total TAG in seed oil.	Durrett et al., 2010
			*C. sativa*	Produced an average of 52% acTAG in seed oil.	Alkotami et al., 2021
	*E. fortunei*	MF06125	*C. sativa*	Produced an average of 72% acTAG in seed oil.	Alkotami et al., 2021
LPAT, DGAT (DAcT)	*C. nucifera* *E. alatus*	Q42670 GU594061	*C. sativa*	Produced about 15% acTAG with MCFA in seeds expressing *UcFATB1* and with silenced endogenous *DGAT1* and *PDAT1*.	Bansal et al., 2018
LPAT2, DGAT1	*C. viscosissima* *C. pulcherrima*	ALM22867 KU055625	*C. sativa*	Enriched MCFA (capric acid, 10:0) in TAG and increased capric acid content to 23.7% of seed oil, which is higher than that in plants expressing these enzymes individually.	Iskandarov et al., 2017
LPAT2, DGAT2	*V. fordii*	MH823254 DQ356682	*A. thaliana*	Increased the content of eleostearic acids to nearly 30% of seed oil in the *fad3/fae1* mutant expressing *VfFADX*.	Shockey et al., 2019
LPAT2, DGAT2	*R. communis*	EU591533 EU391592	*A. thaliana*	Produced a higher level of hydroxy FA (up to 30% of seed oil) in the *fae1* mutant expressing *RcFAH12*, compared to expressing *RcLPAT2* or *RcDGAT2* alone.	Shockey et al., 2019
GPAT9, LPAT2, DGAT2	*R. communis*	EU391594 EU591533 EU391592	*A. thaliana*	Adding *RcGPAT9* to the combination of *RcLPAT2* and *RcDGAT2* did not further increased hydroxy FA content.	Shockey et al., 2019
GPAT9, LPAT2, PDAT1A	*R. communis*	NP_001310690 NP_001310679 NM_001323733	*A. thaliana*	Produced tri-hydroxy-TAG, increased hydroxy FA to 34% of seed oil, and restored seed oil content to wild-type level when co-expressed with *RcFAH12* in the *fae1* mutant.	Lunn et al., 2019
DGAT2, LPCAT, PDAT1-2, PDCT	*R. communis*	EU391592 KC540908 NM_001323733 EQ973818	*A. thaliana*	Produced hydroxy FA to approximately 25% and 31% of seed oil in the wild-type and *fae1* backgrounds, respectively, when co-expressed with *RcFAH12*.	Park et al., 2022

N/A, not available.

**Table 3:** List of specialized lipogenic factors involved in LD biogenesis used for lipid engineering in plants.

Lipogenic Factor	Origin Species	Accession No.	Target Species	Effects of Heterologous Expression on Lipid Metabolism	Reference
OLE	*S. indicum*	AAD42942	*S. tuberosum*	Increased TAG contents in leaves and tubers (3.3% TAG of DW) when combined with other lipogenic factors.	Liu et al., 2017
			*N. tabacum*	Increased TAG contents in leaves, stems, and roots when combined with other lipogenic factors.	Vanhercke et al., 2014 Vanhercke et al., 2017
			*S. bicolor*	Increase TAG (8.4% of DW) and total lipid (9.9% of DW) contents in leaves when combined with other lipogenic factors.	Vanhercke et al., 2019a
	*R. communis*	N/A	*A. thaliana*	Increased hydroxy FA from 18% to 22% of seed oil in Arabidopsis expressing *RcFAH12*.	Lu et al., 2006
Cys-OLE	*S. indicum*	N/A	*A. thaliana*	Enhanced the accumulation of lipids in leaves to a higher level compared with the wild-type SiOLE.	Winichayakul et al., 2013
FIT2	*M. musculus*	BAE37420	*A. thaliana*	Increased the number and size of LDs in leaves and enhanced lipid accumulation in both leaves and seeds.	Cai et al., 2017
			*N. benthamiana*	Promoted LD proliferation and increased levels of neutral lipids in leaves.	Cai et al., 2017
FSP27	*M. musculus*	NM_178373	*A. thaliana*	Increased the number and size of LDs in leaves, and elevated lipid contents in seeds.	Price et al., 2020
			*N. benthamiana*	Mediated LD fusion and increased the number and size of LDs in leaves.	Price et al., 2020

N/A, not available.

## Section I. Producing specialized FAs by tailoring FA synthesis and modification

Lipogenic factors involved in FA synthesis and modification determine the diversity of FAs with respect to their carbon chain lengths, degree of unsaturation, and addition of a variety of functional groups, which determine their physical properties and potential industrial uses. Typical FAs found conserved in the plant kingdom range from 16 to 18 carbons in length and contain 0 to 3 double bounds at Δ^9^, Δ^12^ and Δ^15^ positions (i.e., counting relative to the carboxyl group). In contrast to these “common” FAs, some FAs with shorter or longer chain lengths, additional double bonds, double bond(s) at different position(s), or functional groups at specific locations along the carbon chain are found in specific groups of plant species or non-plant organisms, and thus are referred to as specialty FAs. The structural properties of these specialty FAs make them promising feedstocks for biofuels, industrial products and nutraceuticals. To increase the value of plant lipids, engineering strategies involving the heterologous expression of lipogenic factors related to FA synthesis and modification have been developed to produce specialty FAs in both seed and vegetative tissues of domesticated plant species (Park et al., 2021).

In this section, we describe efforts to evaluate enzymes in the FA biosynthesis and modification pathway that are responsible for producing the following well-studied types of specialty FAs. Medium-chain FAs result from the action of FA thioesterases that release acyl chains from acyl carrier protein (ACP). Hydroxy, epoxy and conjugated FAs arise from the action of enzymes that evolved from the Δ^12^-oleic FA desaturase 2 (FAD2) class of integral membrane desaturases (Shanklin and Cahoon, 1998) which act primarily on oleic acid esterified to PC. Omega-7 monounsaturated FAs with a double bound at the ω^7^ position (i.e., counting relative to the methyl end of FAs) can be produced by Δ^9^-acyl-ACP or Δ^9^-acyl-CoA desaturase with high specificity for 16:0-ACP or 16:0-CoA, respectively (Bondaruk et al., 2007; Nguyen et al., 2010). Very-long-chain PUFAs arise from the action of multiple desaturases and elongases, and their engineering represents a tour-de-force in heterologous expression and pathway optimization (Napier et al., 2019). The last example is the addition of a cyclopropyl group across the double bond in oleic acid by cyclopropane synthase, a class of enzymes present in plants and prokaryotes (Bao et al., 2002; Bao et al., 2003). In section II and III, we summarize the approaches to incorporate these specialized FAs into TAGs and subsequently package them into LDs.

### Medium-chain fatty acids

Medium-chain FAs (MCFAs) include FAs of 8-14 carbons in lengths, generated by the hydrolysis of FA from acyl carrier protein between the C8 and C14 stages of elongation *via* variants of FATB with defined chain length specificities (**Figure 1**). Lipids containing MCFAs are naturally produced in palm kernel (*Elaeis guineensis*), coconut (*Cocos nucifera*), and cuphea genus (*Cuphea pulcherrima*, *Cuphea viscosissima*, *Cuphea palustris*, *Cuphea hookeriana*), and these plants derived MCFAs serve as potential feedstocks for jet fuel and industrial products such as cosmetics and detergents (Dyer et al., 2008; Kallio et al., 2014; Park et al., 2021). To engineer the production of MCFAs in oilseed crops, FATB variants that specifically hydrolyze C8-C14 FAs from acyl-ACPs were isolated from California bay (*Umbellularia californica*) and Cuphea and expressed in *Arabidopsis thaliana*, *Camelina sativa*, and *Brassica napus*. Heterologous expression of *U. californica FATB1* produced MCFAs primarily consisting of lauric acid (C12:0) up to 21%, 37%, and 50% of seed oil in Camelina, Arabidopsis, and *B. napus*, respectively (Eccleston et al., 1996; Voelker et al., 1996; Tjellström et al., 2013; Kim et al., 2015b). FATB variants from different Cuphea species showed different efficiencies and substrate chain length specificities when expressed in oilseed plants (**Table 1**). Expression of *FATB2* from *C. hookeriana* could produce MCFAs ranging from C8 to C14 with capric acid (C10:0) as the most abundant species accounted for approximate 12%, 22%, and 40% of total seed lipids in Camelina, Arabidopsis, and *B. napus*, respectively (Dehesh et al., 1996; Tjellström et al., 2013; Kim et al., 2015b). FATB1 from *C. viscosissima* produced 15% MCFAs with chain lengths varying from C8 to C14 in seed oil when expressed in Camelina, whereas FATB2 from *C. palustris* produced only myristic acid (C14:0) to 24% and 39% of seed oil in Camelina and Arabidopsis, respectively (Tjellström et al., 2013; Kim et al., 2015b). Relatively low levels of MCFAs (1.2%-7.5% of seed oil) were detected in seeds of Arabidopsis and Camelina expressing *FATB1*, *FATB3* or *FATB4* from *C. pulcherrima*, compared with FATBs from other Cuphea species. Based on data collected from Camelina, UcFATB1 and CpFATB2 seem to be the most effective FATB variants in producing MCFAs with UcFATB1 preferentially generating lauric acid (C12:0) and CpFATB2 exclusively producing myristic acid (C14:0). Furthermore, co-expression of *ChFATB2* or *CpFATB1* with a Cuphea medium-chain-specific 3-ketoacyl-ACP synthase (KAS4) that catalyzes the condensation of acyl-ACP with malonyl-ACP increased MCFA content by up to 40% in *B. napus* seed oil as compared with that of plants expressing *FATB* alone (Dehesh et al., 1998). Disruption of acyl-ACP synthetase (AAE15/16) that re-activates FAs released from acyl-ACP in Arabidopsis overexpressing Cuphea *FATB* further enhanced MCFA accumulation in seeds (Tjellström et al., 2013). In addition to the FAT-type thioesterases containing two “hotdog” folds (Mayer and Shanklin, 2005), another acyl-ACP thioesterase family, acyl-lipid thioesterase (ALT) with a single “hotdog” fold, is generally present in all classes of plants (Kalinger et al., 2020). Overexpression of Arabidopsis *ALT1* or *ALT4* in Camelina seeds and *Nicotiana benthamiana* leaves yielded C6–C14 MCFA at a relatively lower level (as much as 3.5% of seed oil) compared to the effective FATB isoforms (**Table 1**; Kalinger et al., 2021).

### Hydroxy fatty acids

The hydroxylation of FAs is mediated by the action of FA hydroxylase (FAH), the first functionally divergent FAD2 homolog to be identified (**Figure 1**). That they are mechanistically related is evidenced by reports that as few as four substitutions between desaturase and hydroxylase can interconvert their functionality (Broun et al., 1998; Broadwater et al., 2002). FAs with hydroxyl groups attached to the acyl chain are useful feedstocks for the formulation of plastics and lubricants (Dyer et al., 2008). The major natural source of hydroxy FAs for industrial uses is castor bean (*Ricinus communis*), which accumulates 90% hydroxy FAs (mostly ricinoleic acid, C18:1-OH) in its seed oil. The enzyme responsible for synthesizing hydroxy FAs in castor is FAH12 (van de Loo et al., 1995). Heterologous expression of RcFAH12 in Arabidopsis led to accumulation of hydroxy FAs consisting of primarily ricinoleic acid accounted for up to 19% of seed oil in wild type, *fad2/fae1*, *fae1*, *fad3*, or *fad3/fae1* backgrounds (Broun and Somerville, 1997; Smith et al., 2003). Similarly, wild-type Camelina expressing RcFAH12 produced approximately 15% hydroxy FAs in seed oil (Aryal and Lu, 2018). Expression of *Hiptage benghalensis* hydroxylases *HbFAH12-1* and *HbFAH12-2* in Arabidopsis *fad2/fae1* mutant yielded up to 21% and 18% hydroxy FA, respectively, in seed oil (Zhou et al., 2013). In contrast to the plant derived FAH12, a FAH homolog isolated from a fungal pathogen, *Claviceps purpurea*, produced 25% hydroxy FAs in seed oil when expressed in the Arabidopsis *fad2/fae1* mutant (Meesapyodsuk and Qiu, 2008). While RcFAH12 can effectively produce hydroxy FAs in target plants and most plant engineering strategies to date have used RcFAH12 to synthesize hydroxy FAs, searching for a more effective FAH from other species to further enhance the accumulation of hydroxy FAs in bioengineered crops might be productive. Lesquerella (*Physaria fendleri*), a Brassicaceae species closely related to Arabidopsis and Camelina, produces about 50% of lesquerolic acid (C20:1-OH), an elongated form of ricinoleic acid, in its seed oil (Horn et al., 2016). Thus, Lesquerella represents a promising alternative industrial oilseed for HFA production, and specialized HFA-related factors in Lesquerella represent a promising source for engineering HFA accumulation in other crops (Horn et al., 2016; Chen et al., 2021). A recent study demonstrated the production of *erythro*-9,10-dihydroxystearate, a vicinal diol by an acyl-ACP desaturase variant *via* dioxygenase chemistry (Whittle et al., 2020). The identification of additional genes that are more efficient at vicinal diol production may facilitate large scale production of these compounds that are difficult to synthesize chemically.

### Epoxy FAs

An epoxy group with its oxygen bridging between adjacent carbons of fatty acyl chains conveys unique chemical reactivity useful for the production of plastics, polymers, coatings, and glues. Epoxy FAs are enriched in seed oils of certain plant species belonging to the Asteraceae and Euphorbiaceae families (Cahoon et al., 2002). Interestingly, the biosynthesis of epoxy FAs in different plant species is catalyzed by different classes of epoxygenase (EPX) enzymes. Those responsible for epoxy FA biosynthesis in Asteraceae species such as *Crepis palaestina*, *Stokesia laevis*, and *Vernonia galamensis* are divergent forms of the FAD2 desaturase, whereas epoxygenases in Euphorbiaceae species such as *Euphorbia lagascae* are cytochrome P450 enzymes (Bafor et al., 1993; Liu et al., 1998; Cahoon et al., 2002). Despite the distinction of these two classes of EPX, heterologous expression of these enzymes in plants resulted in accumulation of similar levels of epoxy FAs (mostly vernolic acid, C18:1- Δ^12^-epoxy FA) in seed oils (**Table 1**). Expression of the FAD2-like EPX coding genes from *C. palaestina* and *S. laevis* led to accumulation of approximately 2.4%-8% epoxy FAs in seed oils of Arabidopsis and soybean, and the cytochrome P450-type EPX from *E. lagascae* produced about 8% epoxy FAs in soybean somatic embryos (Singh et al., 2001; Cahoon et al., 2002; Hatanaka et al., 2004; Li et al., 2010). Providing the exogenous CpEPX with more linoleic acid (C18:2) substrate by disrupting *FAD3* and *FAE1* in Arabidopsis increased the levels of epoxy FAs to 8.6% of seed oil (Zhou et al., 2006). Previous studies suggested that heterologous expression of either type of EPX can reduce the accumulation of linoleic acid in target plants probably caused by decreased activity of the endogenous FAD2 enzyme, and co-expression of EPX with a typical FAD2 dramatically enhanced the production of epoxy FAs to 21% of seed oil in Arabidopsis *fad3/fae1* mutant (Singh et al., 2001; Cahoon et al., 2002; Zhou et al., 2006). In addition, epoxy FAs can also be engineered in non-seed tissues. Expression of *ElEPX* in tobacco (*Nicotiana tabacum*) calli produced epoxy FAs accounted for 15% of total lipids (Cahoon et al., 2002). Transient expression of *SlEPX* in petunia (*Petunia hybrida*) leaves or a FAD2-like EPX from *V. galamensis* in *N. benthamiana* leaves resulted in accumulation of 0.5% or 8.7% epoxy FAs in total leaf lipids, respectively (Li et al., 2010; Sun et al., 2022). Moreover, co-expression of *VgEPX* with *VgFAD2* increased the level of epoxy FAs to 13.1% of total lipids in *N. benthamiana* leaves (Sun et al., 2022). Collectively, divergent classes of EPX from different plant species seem to be equally effective in producing epoxy FAs in seeds and providing more linoleic acid by overexpressing a “typical” *FAD2* or disrupting *FAD3* and *FAE1* is critical for further increasing epoxy FA levels.

### Omega-7 unsaturated fatty acids

Omega-7 unsaturated FAs (ω-7 FAs) are potential feedstocks for the production of octene, a high-demand industrial product used for polyethylene production (Nguyen et al., 2010). Some plants (e.g., milkweed [*Asclepias syriaca*] and cat’s claw vine [*Doxantha unguis-cati*]) can naturally produce ω-7 FAs (e.g., palmitoleic acid 16:1Δ^9^ and cis-vaccenic acid 18:1Δ^11^) by Δ^9^-acyl-ACP desaturase (AAD) with high specificity for 16:0-ACP (Cahoon et al., 1997; Cahoon et al., 1998). Heterologous expression of the milkweed 16:0-ACP desaturase in Arabidopsis failed to produce detectable ω-7 FA, while the Doxantha 16:0-ACP desaturase produced approximately 28% and 9% ω-7 FAs in Arabidopsis and Brassica seed oil, respectively (Bondaruk et al., 2007). An AAD variant with high specificity for converting 16:0-ACP to 16:1 Δ^9^-ACP was selected from a pool of randomized mutants of castor AAD (Cahoon and Shanklin, 2000), and expression of this engineered enzyme (Com25) in Arabidopsis seeds resulted in accumulation of ω-7 FAs to 14% of seed oil (Nguyen et al., 2010). Increasing the level of 16:0-ACP by silencing the 16:0-ACP elongase, β-ketoacyl-ACP synthase II (KASII/FAB1), in *fae1* mutant overexpressing Com25 further increased the content of ω-7 FAs to 56% of seed oil. Co-expressing Com25 with two fungal Δ^9^-16:0-CoA desaturases from *Stagonospora nodorum* (SnD9D) and *Aspergillus nidulans* (AnD9D) in fab1/fae1 mutant increased ω-7 FA content to 71% of seed oil by desaturating saturated FAs after transfer from the plastid to the ER (Nguyen et al., 2010). A similar strategy co-expressing Com25 and a Δ^9^-16:0-CoA desaturase from *Caenorhabditis elegans* (FAT5) in Camelina seeds with 16:0-ACP substrate pools increased by silencing genes encoding KASII/FAB1, FAE1, and16:0-ACP thioesterase (FATB) increased ω-7 FAs to 60-65% of seed oil (Nguyen et al., 2015).

### Conjugated fatty acids

The FAD2 desaturases that produce conjugated FAs by converting Δ^9^ and Δ^12^ double bonds to Δ^11^ and Δ^13^ double bonds are designated as FA conjugases (FADX) (**Figure 1**; Cahoon et al., 1999; Dyer et al., 2002). The higher oxidation rates of conjugated FAs relative to typical polyunsaturated FAs make them useful as drying agents in paints and inks. Conjugated FAs can also serve as health supplements as they have been reported to have fat-reducing and anticancer effects in animals (Lee et al., 2002; Dyer et al., 2008; Yuan et al., 2014). Natural sources of conjugated FAs include tung tree (*Vernicia fordii*), *Momordica charantia*, *Impatiens balsamina*, and *Calendula officinalis*, and genes encoding FADX enzymes have been isolated from these plant species and evaluated for their efficacy in producing conjugated FAs in model plants and oilseed crops. Ectopic expression of FADX coding genes from *I. balsamina*, *M. charantia*, and *C. officinalis* in somatic soybean (*Glycine max*) embryos resulted in production of conjugated FAs to approximately 5%, 18%, and 22% of total FAs, respectively (Cahoon et al., 1999). For engineering approaches carried out in Arabidopsis seeds, mutants with FA desaturase 3 (FAD3) and FAE1 disrupted are used to provide more substrates (linoleic acid) for FADX. Arabidopsis *fad3/fae1* mutants expressing *FADX* genes from *V. fordii*, *M. charantia*, and *C. officinalis* accumulated approximately 6%, 13%, and 15% conjugated FAs in seed oil, respectively (Cahoon et al., 1999; Cahoon et al., 2006). Recent attempts to engineer conjugated FAs in plant vegetative tissues by expressing VfFADX in Arabidopsis successfully produced conjugated FAs (eleostearic acid, 18:3, Δ9c, Δ11t, Δ13t) to 2% of total neutral lipids in leaves (Yurchenko et al., 2017). Among all FADX enzymes tested so far, CoFADX seems to be the most effective enzyme for producing high levels of conjugated FAs in both Arabidopsis and soybean, but different FADX orthologs produce different types of conjugated FAs. Conjugated FAs produced by CoFADX comprise exclusively calendic acid (18:3, Δ8t, Δ10t, Δ12c), while eleostearic acid is the primary conjugated FAs detected in transgenic plants expressing *VfFADX* or *McFADX*. In future efforts to engineer conjugated FAs, it will be important to select a FADX that produces high levels and desired types of conjugated FAs.

### Very-long-chain polyunsaturated FAs

Very-long-chain polyunsaturated FAs (VLCPUFA) such as eicosapentaenoic acid (EPA) and docosahexaenoic acid (DHA) are FAs with 20 or 22 carbons and 4 to 6 double bonds. EPA and DHA are valuable nutraceuticals because of their beneficial roles in fetal neuronal system development and cardiovascular diseases prevention (Tocher et al., 2019). Marine fish oils are a major source of EPA, DHA and other ω^3^-VLCPUFAs. However, the marine-sourced EPA and DHA are insufficient to meet the increasing demand for these FAs in human diet (Tocher et al., 2019). To develop sustainable alternative sources of EPA and DHA, in the past two decades, enormous research effort has been directed at engineering ω^3^-VLCPUFAs in plant oils, and significant progress has been achieved in producing EPA and DHA in oilseed crops (Napier et al., 2019). There are two pathways that can produce VLCPUFAs from linoleic acids: 1) the conventional pathway including Δ^6^-desaturase (DES), Δ^6^-elongase (ELO), Δ^5^-DES, Δ^5^-ELO, and Δ^4^-DES; and 2) the alternative pathway using Δ^9^-ELO, Δ^8^-DES, Δ^5^-DES, Δ^5^-ELO, and Δ^4^-DES. Both pathways have been introduced into plants and successfully produced EPA and DHA in both seed and vegetative tissues (Qi et al., 2004; Wu et al., 2005; Wood et al., 2009; Petrie et al., 2010). It was later found that, besides the minimum five enzymes required for DHA biosynthesis in plants, introduction of a highly active Δ^12^-DES from *Lachancea kluyveri* and a Δ^15^/ω^3^-DES with broad substrate specificity from *Pichia pastoris* could effectively increase the ratio of ω^3^/ω^6^ FAs and therefore the level of DHA in seed oil (Petrie et al., 2012; Petrie et al., 2014). To optimize the engineering strategy to produce high levels of EPA and DHA in plants, DES and ELO enzymes from a wide variety of species including algae, fungi, oomycetes, mosses, animals, and flowering plants were introduced into plants to test their efficacy in producing EPA and DHA in various plant species (Abbadi et al., 2004; Qi et al., 2004; Wu et al., 2005; Wood et al., 2009; Petrie et al., 2010; Petrie et al., 2012; Petrie et al., 2014; Han et al., 2020; Han et al., 2022). The optimal enzyme combination that achieved the highest levels of EPA and DHA (over 20%) in seed oil reported to date consists of a Δ^12^-DES from *Phytophthora sojae*, Δ^15^/ω^3^-DES from *Phytophthora infestans*, Δ^6^-DES from *Ostreococcus tauri*, Δ^6^-ELO from *Physcomitrella patens*, Δ^5^-DES from *Thraustochytrium* sp., Δ^5^-ELO from *O. tauri*, and Δ^4^-DES from *Ostreococcus* RCC809 (Han et al., 2020; Han et al., 2022).

### Cyclopropane fatty acids

Cyclopropane FAs (CPAs), such as dihydrosterculic acid (9, 10-methylene octadecanoic acid) and lactobacillic acid (11, 12 methylene octadecanoic acid), are specialized FAs that contain a cyclopropane group (three-carbon carbocyclic ring) within the carbon chain. They are found in bacteria and certain plant species such as *Litchi chinensis*. The highly strained and reactive carbocyclic ring of CPA readily opens to form methyl-branched fatty acids, which exhibit unique physical and chemical properties such as low melting temperatures, resistance to oxidation, and propensity for self-polymerization, making CPAs suitable for application in lubricants, paints, and coatings (Carlsson et al., 2011). Ectopic expression of genes encoding cyclopropane synthase (CPS), the enzyme catalyzes the conversion of monounsaturated FAs to CPAs (**Figure 1**), successfully produced CPAs in plants that normally lack these compounds. Intriguingly, the CPS gene from *Escherichia coli* is more effective than CPS homologs isolated from plant species for synthesizing CPAs in plants (**Table 1**). Engineering of CPAs in Arabidopsis and *Camelina* seeds were carried out in mutant lines with reduced FA desaturase 2 (FAD2) and FA elongase 1 (FAE1) that accumulate increased levels of oleoyl substrates for CPA production. Expression of *CPS* genes cloned from *Sterculia foetida* and cotton (*Gossypium hirsutum*) in Arabidopsis *fad2/fae1* mutant lines led to the accumulation of CPA to between 0.05% and 1% of total seed FAs, respectively (Yu et al., 2011). In contrast, the expression of *E. coli* CPS resulted in nearly 10% CPA accumulation in seed oil in Arabidopsis and Camelina (Yu et al., 2014; Yu et al., 2018). Recently, researchers tested the ability of CPS enzymes to synthesize CPAs in plant vegetative tissues by transiently expressing cotton or *E. coli CPS* genes in *N. benthamiana* leaves and found that GhCPS1 produced up to 1% CPA of total leaf FAs while *EcCPS* expression led to the accumulation of CPA up to 3.7% of total lipids (Okada et al., 2020). The levels of CPAs in leaf lipids can be further elevated to 4.8% and 11.8% by silencing the expression of endogenous *NbFAD2* in leaves expressing *GhCPS1* and *EcCPS*, respectively (Okada et al., 2020). Therefore, future strategies to engineer CPA accumulation in plant seed and vegetative tissues will exploit EcCPS rather than plant CPS variants.

## Section II. Optimizing lipid accumulation by channeling selected FA toward TAG

TAGs, also known as storage lipids, are the most abundant form of vegetable oils. They primarily accumulate in seeds to provide energy for seed germination and establishment. In plant vegetative tissues such as leaves, TAGs are barely detectable and serve primarily as transient intermediates for FAs removed from membrane lipids prior to their degradation (Xu and Shanklin, 2016). Accumulation of free FAs in cells and specialty FAs in membrane lipids can result in negative effects on plant growth. One reason for this is that they elicit feedback inhibition of FA synthesis *via* biotin attachment domain-containing protein (BADC), a negative regulator of ACCase (Yang et al., 2015; Salie et al., 2016; Zale et al., 2016; Keereetaweep et al., 2018; Yu et al., 2021). Channeling FA flux toward the TAG pool can reduce the accumulation of free FAs and remove specialty FAs from membrane lipids, thereby mitigating their negative growth effects and further enhancing the accumulation of lipids with desired acyl composition. Therefore, Lipogenic factors involved in glycerolipid assembly capable of accommodating specialty FA substrates represent critical targets for enhancing specialty FA-containing TAG accumulation in plants. In the following subsections, we summarize previous efforts to assess the efficacies of TAG-assembly-related enzymes for optimizing the accumulation of desired lipids.

### Incorporating specialty FA into TAG

Incorporation of specialty FA into TAG requires specialized enzymes that can recognize the specialized FA for catalyzing multiple steps of TAG assembly (**Figure 1**). In the glycerolipid biosynthesis pathway, The ER-localized LPAT catalyzes the transfer of FAs from the acyl-CoA pool to LPA to form PA, which serves as a key intermediate for channeling FAs into TAGs and membrane lipids. Lysophosphatidylcholine acyltransferase (LPCAT) incorporates FAs into PC. PDCT and PLC catalyze the conversion of PC to DAG and thereby allow FAs esterified to PC to enter the DAG pool, which are subsequently converted to TAG by DGAT and PDAT. Below we describe some variants of these enzymes that are specialized for incorporating different types of specialty FAs into TAGs.

#### LPATs with substrate specificities for specialty FAs

Divergent LPATs from specialty FA-accumulating organisms have evolved specialized substrate specificities for incorporating specialty FAs into TAGs (**Table 2**). For instance, LPAT variants from organisms naturally accumulating MCFAs (e.g., *C. nucifera*, *C. viscosissima*, and *C. pulcherrima*) preferentially incorporate MCFAs to the *sn*-2 position of LPA, and when combined with FATBs, enabled efficient deposition of MCFAs at the *sn*-2 position of TAG and further increased total MCFA contents (Knutzon et al., 1999; Kim et al., 2015a; Kim et al., 2015b). Notably, expression of *CnLPAT*, *CvLPAT2*, and *CpuLPAT2a* resulted in the deposition of capric acid (C10:0) at the TAG *sn*-2 position, whereas *CpuLPATB* expression led to accumulation of myristic acid (C14:0) instead of capric acid (C10:0) at the *sn*-2 position of TAG, suggesting distinct substrate specificities of divergent forms of LPATs for different MCFAs (Knutzon et al., 1999; Kim et al., 2015a; Kim et al., 2015b). RcLPAT2 isolated from castor and VfLPAT2 from tung tree producing conjugated FAs improved the accumulation of hydroxy FAs and conjugated FAs, respectively, in Arabidopsis seeds (Shockey et al., 2019). Co-expression of the *LPAT* from epoxy FA-rich *V. galamensis* with *VgEPX* increased the level of epoxy FAs from 8.7% (*VgEPX* alone) to 16.7% of total lipids in *N. benthamiana* leaves (Sun et al., 2022). For CPA engineering, co-expression of *SfLPAT2* from CPA-enriched *S. foetida* with *EcCPS* in the *fad2/fae1* mutant resulted in the accumulation of CPA at both *sn*-1 and *sn*-2 positions of PC and further increased CPA contents to 35% and 18% of seed oils in Arabidopsis and Camelina, respectively (Yu et al., 2014; Yu et al., 2018).

#### LPCAT, PDCT and PLC variants channeling specialty FA into PC and DAG

Given the crucial role of PC in acyl editing and TAG biosynthesis, specialized LPCAT, the enzyme catalyzing the conversion of lysophosphatidylcholine (LPC) and acyl-CoA to PC, may contribute to the incorporation of specialty FAs into TAGs. Indeed, the specialized LPCAT from *V. galamensis*, an oleaginous plant containing high levels of epoxy FAs in its seed oil, greatly enhanced the accumulation of epoxy FAs from 8.7% to as much as 19.4% of total lipids when co-expressed with *VgEPX* in *N. benthamiana* leaves (Sun et al., 2022). Studies of transgenic Arabidopsis and Camelina engineered to produce CPA and hydroxy FAs revealed that PCs containing these specialty FAs were not efficiently converted to DAGs and TAGs, identifying bottlenecks for the accumulation of specialty FAs (Bates and Browse, 2011; Yu et al., 2018). To address these bottlenecks, specialized enzymes that convert CPA-containing or hydroxy-containing PCs to DAGs including PDCT and PLC were used to further enhance the production of specialty FAs (**Figure 1**; **Table 2**). In efforts to engineer hydroxy FAs in model and crop plants, a PDCT (RcPDCT) and a PLC (RcPLCL1) were isolated from castor and tested in transgenic plants expressing *RcFAH12*. It was shown that both RcPDCT and RcPLCL1 could enrich hydroxy FA in DAG and TAG and increase total hydroxy FA contents from 10-15% to approximately 20% of seed oil (Hu et al., 2012; Aryal and Lu, 2018). Co-expression of *LcPDCT* from CPA-enriched *L. chinensis* with *EcCPS* enhanced the deposition of CPA in DAG and TAG and led to a 50% increase of CPA in seed oil compared with that of plants expressing *EcCPS* alone (Yu et al., 2019).

#### Specialized DGATs and PDATs for producing TAGs containing specialty FAs

Several studies have reported that specialized DGATs and PDATs with high specificities for specialty FAs are necessary for addressing bottlenecks for the accumulation of specialty FAs in target plants (Park et al., 2021; Lunn et al., 2022). In one of such study, a DGAT from *C. pulcherrima*, namely CpuDGAT1 was identified, which showed a higher enzyme activity toward MCFA substrates relative to typical FAs. Expression of *CpuDGAT1* in Camelina seeds containing MCFA produced by the exogenous CvFATB1 enriched MCFA (capric acid, 10:0) in TAG and increased capric acid content from 8% to 14.5% of seed oil (Iskandarov et al., 2017). Two DGATs from *V. galamensis* were tested in petunia leaves and soybean seeds for their ability to enhance epoxy FA accumulation. When co-expressed with the epoxygenase gene from *Stokesia laevis* (*SlEPX*), both *VgDGAT1* and *VgDGAT2* further increased epoxy FA contents in petunia leaves and soybean seeds, and VgDGAT2 seemed to have a greater impact on epoxy FA accumulation than VgDGAT1 (Li et al., 2010). To engineer conjugated FAs in Arabidopsis, *VfDGAT2*, a DGAT gene isolated from tung tree was co-expressed with *VfFADX*. While no significant increase in eleostearic acid was detected in seeds co-expressing *VfDGAT2* and *VfFADX* relative to that in seeds expressing *VfFADX* alone, introducing *VfDGAT2* into Arabidopsis leaves expressing *VfFADX* resulted in redirection of eleostearic acids from phospholipids to TAGs, an increase in eleostearic acid contents, and mitigation of negative growth effects (Yurchenko et al., 2017; Shockey et al., 2019). For hydroxy FA engineering, specialized DGAT and PDAT isolated from *R. communis* were combined with RcFAH12 individually to produce TAGs with high levels of hydroxy FAs, and both RcDGAT2 and RcPDAT1A enhanced the incorporation of hydroxy FAs into TAG and increased the content of hydroxy FAs to 30% and 27% of seed oil, respectively (Burgal et al., 2008; van Erp et al., 2011; Shockey et al., 2019).

#### Combinations of TAG-assembly enzymes to enrich specialty FA in TAG

To further enhance the production of specialty TAGs, enzymes involved in different steps of TAG assembly were combined to maximize the incorporation of the specialty FAs into TAGs (**Table 2**). Combining CvLPAT2 and CpuDGAT1 from MCFA-enriched Cuphea species greatly enriched capric acid (C10:0) accumulation in TAG and increased capric acid content to 23.7% of seed oil, which is higher than that in Camelina expressing these enzymes individually (Iskandarov et al., 2017). For hydroxy FA engineering, RcLPAT2 and RcDGAT2 isolated from castor synergistically increased the level of hydroxy FA to up to 30% of seed oil, but adding RcGPAT9, a specialized GPAT that incorporates hydroxy FAs to the *sn*-1 position of G3P, to this combination did not further boost the accumulation of hydroxy FAs (Shockey et al., 2019). In a similar study, Lunn et al. (2019) successfully enriched tri-hydroxy-TAG, increased hydroxy FA to 34% of seed oil, and restored seed oil content to wild-type levels by co-expressing *RcGPAT9*, *RcLPAT2*, *RcPDAT1A* in an *RcFAH12* transformed Arabidopsis *fae1* mutant (**Table 2**). Another combination including RcLPCAT, RcPDCT, RcPDAT1-2, RcDGAT2 produced about 31% hydroxy FA in Arabidopsis *fae1* mutant seeds expressing RcFAH12 and increased both seed size and oil per seed (Park et al., 2022).

### Producing acetyl-TAG using specialized DGATs

Acetyl-TAGs (acTAGs) are specialty TAGs with an acetate esterified to the *sn*-3 position in place of a long-chain fatty acid. Oils containing acTAGs exhibit reduced viscosity and therefore have high value in a wide variety of industrial applications such emulsifiers and lubricants. Specialized DGATs (DAcT) responsible for acTAG biosynthesis were isolated from *Euonymus alatus* and *Euonymus fortune*, plants that naturally produce acTAGs in their seeds. Heterologous expression of *EaDAcT* resulted in accumulation of 40% and 52% of acTAG in seeds of Arabidopsis and Camelina, respectively (Durrett et al., 2010). EfDAcT, functioning more efficiently than EaDAcT, produced an average of 72% acTAG in transgenic Camelina seeds (Alkotami et al., 2021). Interestingly, co-expression of *CnLPAT* from MCFA-containing coconut and *EaDAcT* from acTAG-enriched *E. alatus* in camelina plants expressing *UcFATB1* produced acTAGs with MCFAs, which have yet to be found in nature, suggesting a potential synthetic-biology strategy for creating novel lipid structures in plants (Bansal et al., 2018).

### Enhancing TAG accumulation by introducing an effective DGAT

DGAT enzymes catalyze the final committed step of TAG biosynthesis, and an efficient DGAT is key to enhancing TAG accumulation in plants (**Figure 1**). Zienkiewicz et al. (2017) screened six out of 12DGATs from *Nannochloropsis oceanica*, a microalga that produces high amounts of TAGs, and identified DGAT5 (DGTT5) as the most efficient isoform for restoring TAG synthesis in a TAG synthesis-deficient mutant of yeast. Transient expression of *NoDGTT5* in *N. benthamiana* leaves led to a 2-fold increase in TAG, and stable expression of *NoDGTT5* in Arabidopsis increased leaf TAG contents by 6-fold and boosted seed oil content by 50% (Zienkiewicz et al., 2017). In another study of DGATs from microalga, DGAT1 from *Chlorella ellipsoidea* increased the oil content by 8–37% and by 12–18% in seeds of Arabidopsis and *B. napus*, respectively (Guo et al., 2017). In addition, mouse (*Mus musculus*) DGAT2, the predominant DGAT responsible for TAG biosynthesis in mouse, when transiently expressed in *N. benthamiana* leaves, produced over 20-fold more TAG than that of control leaves (Cai et al., 2019). Recently, a study of *Cyperus esculentus*, a unique plant accumulating large amounts of TAG in its underground tubers, revealed that its heterologous expression in *N. tabacum* increased the TAG content to 5.5% of leaf dry weight (DW), which is 7.2-fold and 1.7-fold higher than that in wild-type leaves and leaves expressing *AtDGAT1*, respectively (Gao et al., 2021). Moreover, *CeDGAT2-2* expression resulted in a substantial increase in the proportion of oleic acid in *N. tabacum* leaves (Gao et al., 2021). In another study, heterologous expression of Arabidopsis *DGAT1* reportedly led to a 7-fold increase in TAG contents in *N. tabacum* leaves (Bouvier-Navé et al., 2000). That the DGATs tested in different studies were driven by different promoters, expressed either transiently or stably, and tested in different plant tissues and species, precludes us from making meaningful comparisons for assessing the relative efficacy of DGATs from different sources. Thus, it would be useful to evaluate all promising DGATs under same conditions and in same tissues and target organisms.

## Section III. Packaging storage lipids into lipid droplets and reducing degradation

It has been demonstrated in Arabidopsis that FA degradation proceeds *via* a TAG intermediate (Fan et al., 2014). Emerging evidence indicates that proper and efficient packaging of TAGs into LDs is critical for increasing the capacity of lipid accumulation in plant cells, and some lipogenic factors involved in this process have been included in metabolic engineering strategies to enhance lipid production in plants (**Table 3**). In this section, we describe some attempts to enhance specialty lipid accumulation in plants with the use of LD-related factors and list other LD-related factors that could be engineered for specialty lipid accumulation in plants.

Oleosins (OLE), the predominant LD coat proteins specific to plants, have been used in several studies to engineer LDs for increased lipid accumulation in plant cells. The L-oleosin from sesame (*Sesamum indicum*) and especially its modified version (cysteine- [Cys]-oleosin) have been combined with other lipogenic factors to engineer storage lipids in vegetative tissues of Arabidopsis, *N. tabacum*, *Solanum tuberosum*, and *Sorghum bicolor* (Winichayakul et al., 2013; Vanhercke et al., 2014; Vanhercke et al., 2017; Liu et al., 2017; Vanhercke et al., 2019a). Expression of the castor *RcOLE* in *RcFAH12*-expressing Arabidopsis further increased hydroxy FA from 18% to 22% of seed oil (Lu et al., 2006).

SEIPIN, a key protein that orchestrates the machinery of LD biogenesis at the ER, can promote LD biogenesis and increase TAG contents in plants (Cai et al., 2015). Overexpression of *AtSEIPIN1* in Arabidopsis seeds engineered to synthesize hydroxy FAs increased the hydroxy FA and total lipid contents, representing a potential new target for engineering specialty FAs in plants (Lunn et al., 2018). Interestingly, some LD proteins without apparent homologs in plants still exhibit conserved functional features as part of the LD biogenesis machinery when ectopically expressed in plants and thus can be used as tools to manipulate LD formation in plants. For instance, ectopic expression of the mouse fat storage-inducing transmembrane protein 2 (FIT2), an ER-localized protein that facilitates the portioning of TAGs from the ER into nascent LDs, in Arabidopsis and *N. benthamiana* led to increased numbers and sizes of LDs and enhanced lipid accumulation in both leaves and seeds (Cai et al., 2017). In another similar study, mouse fat-specific protein 27 (FSP27), a vertebrate-specific protein that mediates LD fusion, was found to promote LD fusion, and enhance the accumulation of LDs and TAGs when expressed in Arabidopsis and *N. benthamiana* (Price et al., 2020). It is an open question whether proteins related to LD formation have evolved specificities for packaging selected specialty TAGs into LDs. Future efforts to elucidate the roles of LD-related proteins in specialty FA accumulation will shed new light on metabolic engineering of desirable lipids in plants.

LD-associated lipases hydrolyze TAGs to release FAs, which are subsequently catabolized *via* β-oxidation in the peroxisomes to produce acetyl-CoA (Eastmond and Graham, 2001). SUGAR DEPENDENT 1 (SDP1) is a primary TAG lipase responsible for TAG degradation in plants (Eastmond, 2006; Kelly et al., 2013b). The suppression of *SDP1* during seed development resulted in increased production of seed oil in Arabidopsis (van Erp et al., 2014), *B. napus* (Kelly et al., 2013a), *Jatropha curcas* (Kim et al., 2014), and soybean (Kanai et al., 2019; Aznar-Moreno et al., 2022). SDP1 from *Physaria fendleri* has been shown to preferentially hydrolyze TAGs containing hydroxy FAs and suppression of its expression increased total FA content by 14-19%, primarily contributing to the significantly increased hydroxy FA (Azeez et al., 2022). Recent studies identified additional proteins involved in the mobilization of LDs in plants including UBX-domain containing protein 10 (PUX10), CELL DIVISION CYCLE 48, (CDC48A), Comparative Gene Identification-58 (CGI58), ATP-binding cassette transporter-like protein (PXA1), and AT-hook motif containing nuclear localized transcriptional repressor (AHL4) (Zolman et al., 2001; James et al., 2010; Park et al., 2013; Deruyffelaere et al., 2018; Kretzschmar et al., 2018; Cai et al., 2020). Future work to tune the expression of these factors may contribute further to enhancing the accumulation of lipids, including specialty FAs, in plants.

## Concluding remarks and future perspectives

Extensive efforts and substantial progress have been made in the past two decades to design and test metabolic engineering strategies for producing desirable lipids in plants for bioenergy, industrial, and nutraceutical purposes. These studies have generated a broad array of lipogenic factors for engineering different types of lipids in various plant species. Selecting lipogenic factors that outperform their alternatives when expressed in a target crop is key to optimizing the design of engineering approaches for maximized production of selected lipids. Notably, the optimal lipogenic factors for plant lipid engineering may be sourced outside of the plant kingdom. For instance, the CPS from *E. coli* and the FAH from a fungal pathogen (*C. purpurea*) were shown to be more effective in producing CPA or hydroxy FA in plants than the plant-sourced ones (Meesapyodsuk and Qiu, 2008; Yu et al., 2014). In organisms producing high levels of specialty FAs, besides the enzymes responsible for FA synthesis and modification, other lipogenic factors function in glycerolipid assembly and LD formation may have evolved specialized features to accommodate these specialty FAs by depositing them in TAGs and subsequently packaging them in LDs. Therefore, future efforts to enhance specialty lipid accumulation in agronomic crops may be enhanced by introducing multiple specialized lipogenic factors involved in all key steps in lipid synthesis and packaging.

As our understanding of the structural basis of specialized lipogenic factors increases, future research of metabolic engineering will benefit from designing novel lipogenic factors that can outperform naturally occurring ones or produce novel lipids that have not been previously identified in nature based on sequence comparison, computational protein design or directed evolution. Deployment of new computational tools such as AlphaFold to these efforts will likely enhance their success (Mirdita et al., 2022). The feasibility of the former approach has been validated in several studies. In attempts to generate novel DGAT enzymes with improved efficiencies in TAG production, mutant variants of soybean and hazelnut (*Corylus americana*) DGAT1s produced higher levels of TAGs when expressed in plants compared to the wild-type versions (Roesler et al., 2016; Hatanaka et al., 2022). The structural details of acyl-ACP desaturases guided the generation of a mutant Δ9-acyl-ACP with amino acid substitutions in the substrate binding pocket, which was combined with other lipogenic factors to engineer the specialty ω^7^ monounsaturated FAs in seed oil (Cahoon and Shanklin, 2000; Nguyen et al., 2010) (Whittle and Shanklin, 2001). Similarly, expression of the native *M. charantia* FADX in Arabidopsis *fad3/fae1* mutant yielded 10% α-eleostearic acid, while the mutagenized McFADX (G111V) or McFADX (G111V/D115E) resulted in a doubling of conjugated FA accumulation to approximately 20% of seed oil. Like the native McFADX, the mutant McFADX (G111V) produced predominantly α-eleostearic acid and little punicic acid, whereas the McFADX (G111V/D115E) double mutant produced nearly equal amounts of α-eleostearic acid and punicic acid (Rawat et al., 2012). In addition, variants of the castor stearoyl-ACP desaturase (T117R/D280K) generated by site-directed mutation can synthesize a novel FA, *erythro*-9,10-dihydroxystearate, with vicinal hydroxyl groups at C9 and C10 positions (Whittle et al., 2020). Improved mechanistic understanding will facilitate the development of novel improved lipogenic factors *via* site-directed mutagenesis i.e., rational, structure-based design in combination with computational modeling (Guy et al., 2022) that can be optimized by design-build-test-learn cycles for plant lipid engineering.

Whereas the majority of plant lipid metabolic engineering has focused on seeds, there is growing interest in engineering lipids in plant vegetative tissues because of their high biomass and high capacity for FA synthesis. Most of the FA flux in plant vegetative tissues is for phospholipids to support membrane synthesis, while TAGs serve as an intermediate for FA degradation and are present only at a minimal level in vegetative tissues (Fan et al., 2014). A variety of lipogenic factors and combinations thereof, have been evaluated for their efficacies in enhancing storage lipid accumulation in vegetative tissues of a small number of plant species (Vanhercke et al., 2019b). So far, the most successful approach, characterized as the “push, pull, and protect” strategy, include 1) seed-specific transcription factors such as WRINKLED1 and LEAFY COTYLEDON2 (LEC2) to push the carbon flux toward FA synthesis, 2) acyltransferases such as DGAT and PDAT to pull FAs into the TAG pool, and 3) LD proteins such as oleosin to package TAGs into LDs and protect them from degradation (Vanhercke et al., 2014; Zale et al., 2016; Vanhercke et al., 2017; Alameldin et al., 2017; Liu et al., 2017; Vanhercke et al., 2019a). A major challenge for enhancing TAG accumulation in non-seed tissues is the impairment of growth associated with TAG accumulation, which may result from the accumulation of cytotoxic free FAs, toxic effects of expression of seed-specific transcription factors, and/or the enlarged TAG pool redirecting carbon flux away from other metabolic pathways (Yang et al., 2015; Zale et al., 2016; Vanhercke et al., 2019a; Mitchell et al., 2020). Future efforts to develop improved strategies for mitigated growth impairment and further enhancement of vegetative TAG production will focus on the identification of alternative lipogenic factors that can more efficiently incorporate FAs to TAGs and have reduced negative impacts on plant growth. Additional promising approaches include restricting the expression of lipogenic factors to certain tissues or growth stages using inducible or tissue-specific promoters (Andrianov et al., 2010; Kim et al., 2015c; Liang et al., 2022) , or the expression of factors such as purple acid phosphatase2 (Cai et al., 2022). Despite the challenges, vegetative biomass represents a sustainable and economical platform for lipid accumulation and the success in engineering TAG accumulation therein will facilitate increased yields per unit land area of high-value lipids containing specialty FAs in vegetative tissues by introducing additional specialized lipogenic factors.

## Data availability statement

The original contributions presented in the study are included in the article/supplementary material. Further inquiries can be directed to the corresponding authors.

## Author contributions

X-HY, YC, and JS conceived the study; YC and X-HY drafted the manuscript; JS revised the manuscript. All authors contributed to the article and approved the submitted version.
